# Fetal MRI Biomarkers and the Prenatal Origins of Autism Spectrum Disorder: A Narrative Review

**DOI:** 10.3390/jcm15093502

**Published:** 2026-05-03

**Authors:** Mariarosaria Motta, Laura Sarno, Dario Colacurci, Daniela Terracciano, Silvia Visentin, Erich Cosmi, Camilla Grelloni, Andrea Ciavattini, Stefano Raffaele Giannubilo, Giuseppe Maria Maruotti

**Affiliations:** 1 Department of Public Health, University of Naples Federico II, 80131 Naples, Italy; mariarosariamotta1@gmail.com (M.M.); dario.colacurci@unina.it (D.C.); giuseppemaria.maruotti@unina.it (G.M.M.); 2 Department of Neurosciences, Reproductive Science and Dentistry, University of Naples Federico II, 80131 Naples, Italy; laurettasarno@gmail.com; 3 Department of Translational Medical Sciences, School of Medicine, University of Naples Federico II, 80131 Naples, Italy; daniela.terracciano@unina.it; 4 Maternal Fetal Medicine Unit, Department of Women’s and Children’s, School of Medicine, University of Padua, 35128 Padua, Italy; silvia.visentin.1@unipd.it (S.V.); erich.cosmi@unipd.it (E.C.); 5 Department of Clinical Sciences, Polytechnic University of Marche, 60123 Ancona, Italy; c.grelloni@pm.univpm.it (C.G.); a.ciavattini@univpm.it (A.C.)

**Keywords:** autism spectrum disorder, fetal MRI, prenatal neurodevelopment, functional connectivity, volumetric biomarkers, insular cortex, ventriculomegaly, tuberous sclerosis complex

## Abstract

**Objectives**: Autism spectrum disorder (ASD) is increasingly conceptualized as a neurodevelopmental condition with prenatal origins. Advances in fetal magnetic resonance imaging (MRI), including high-resolution structural imaging and resting-state functional connectivity analysis, now enable in vivo characterization of the developing human brain before birth. This review examines whether fetal MRI biomarkers are associated with later ASD diagnosis or autistic traits. **Methods**: We conducted a PRISMA-informed narrative review of human studies identified through MEDLINE, EMBASE, SCOPUS, and Web of Science. Eligible studies included original human investigations using fetal MRI to assess brain structure and/or function, with postnatal ASD diagnosis or standardized autistic-trait outcomes. **Results**: Eight eligible studies provide converging evidence that neurodevelopmental divergence associated with ASD may be detectable in utero. Structural analyses consistently report prenatal volumetric alterations, particularly enlargement of the insular cortex between the second and third trimesters. Additional findings of regional overgrowth and hemispheric asymmetries suggest distributed deviations in cortical maturation. Functional fetal MRI studies further demonstrate atypical large-scale network organization prior to birth. Altered connectivity within cingulate, prefrontal, temporal, and cerebellar circuits has been prospectively associated with later autistic traits, indicating that network-level integration may diverge before behavioral symptoms emerge. Evidence from high-risk conditions, including isolated ventriculomegaly and tuberous sclerosis complex, reinforces the association between prenatal structural abnormalities and increased ASD risk. **Conclusions**: Current evidence suggests that structural and functional brain alterations identifiable by fetal MRI may precede the clinical manifestation of ASD. These findings support a model of ASD as a condition potentially rooted in prenatal neurodevelopmental divergence. However, larger, standardized, multicenter studies are required before fetal MRI biomarkers can be translated into predictive or clinical applications.

## 1. Introduction

Autism spectrum disorder (ASD) is a complex neurodevelopmental condition characterized by persistent social and communication challenges, as well as restricted interests and repetitive behaviours. ASD prevalence is estimated at approximately 1–2% among children globally. While diagnosis typically occurs in early childhood, accumulating evidence indicates that ASD emerges during prenatal brain development [1,2].

Genetic research has highlighted intricate polygenic influences, while neuropathological studies of postmortem ASD brains have uncovered fundamental abnormalities in neuronal migration, focal cortical dysplasia, and disrupted laminar organization. Furthermore, large-scale neuroimaging initiatives, such as the ENIGMA Working Groups, have documented consistent variations in cortical thickness and regional brain volumes in individuals with ASD compared to neurotypical controls [3]. These macroscopic changes are widely interpreted as potential markers of deeper disruptions in early neurodevelopmental milestones, including neuronal proliferation, migration, synaptogenesis, and the initial formation of neural networks [4,5].

Although ASD is widely recognized as a disorder of early development, most neuroimaging research has focused on postnatal cohorts. The prenatal period, during which crucial events such as cortical patterning, gyrification, and the establishment of large-scale neural networks occur, has been less studied, primarily due to technical challenges inherent to fetal imaging.

Recent advancements in fetal MRI have accelerated research in this field. High-resolution structural imaging now enables measurement of fetal brain structures as early as the second trimester. Simultaneously, resting-state functional MRI (rs-fMRI) permits mapping of early functional brain networks in utero. These methods provide new opportunities to determine whether neuroanatomical differences characteristic of ASD are apparent prior to birth.

This review synthesizes the current human evidence exploring the link between fetal MRI biomarkers and subsequent ASD diagnoses or autistic traits. By merging data from structural, functional, and high-risk cohort studies, we aim to determine whether prenatal neuroimaging can shed light on the pathogenesis of ASD and provide early markers of neurodevelopmental risk.

## 2. Materials and Methods

### 2.1. Search Strategy and Information Sources

This manuscript should therefore be interpreted as a PRISMA-informed narrative review rather than as a formal systematic review or scoping review.

PRISMA was used to improve transparency in study identification and selection, whereas the synthesis remained narrative.

In line with the attached PRISMA checklist, the title identifies the article as a review, the search strategy and study selection are explicitly reported, and risk-of-bias assessment is marked as not applicable because no formal systematic review methodology or pooled quantitative synthesis was undertaken.

To ensure a rigorous and transparent methodology, a structured literature search and study selection process were conducted using PRISMA 2020 as a reporting framework for identification, screening, and transparent study selection;

However, the review was designed as a narrative review rather than as a formal systematic review or meta-analysis.

This framing is consistent with the accompanying PRISMA checklist, in which the review is identified as a review and the risk-of-bias item is reported as not applicable because of the narrative design. We queried four major academic databases: MEDLINE, Embase, Scopus, and Web of Science. The search encompassed all records from database inception through January 2026, with no language restrictions to maximize the identification of relevant research. To supplement the electronic search, we utilized “backward citation tracking,” manually screening the reference lists of key papers and eligible studies to uncover additional records that might have been missed in the initial query. The full electronic search strategy is reported in Appendix A.

The search strategy employed combinations of several key terms, including “fetal,” “prenatal,” or “in utero,” paired with “magnetic resonance imaging” or “MRI,” and “autism,” “autism spectrum disorder,” “ASD,” or “autistic traits.”

### 2.2. Study Selection Process

**Figure 1:**
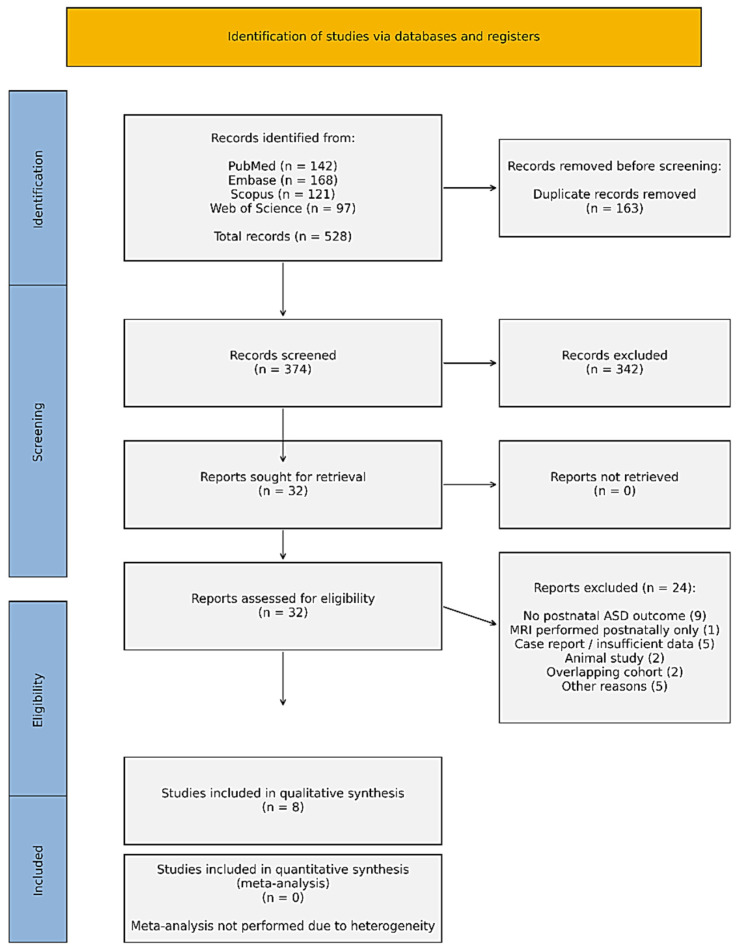
Flowchart of included study.

The complete selection process is visually detailed in the PRISMA 2020 flow diagram (Figure 1). Our initial multi-database search yielded a total of 528 records (Medline *n* = 142; Embase *n* = 168; Scopus *n* = 121; Web of Science *n* = 97). After removing 163 duplicate entries, 374 unique records were screened based on their titles and abstracts. During this preliminary phase, 342 records were excluded as they failed to meet the core inclusion criteria, most frequently due to a lack of fetal MRI data or the absence of postnatal ASD-related outcomes. Of the remaining 32 articles retrieved for full-text eligibility assessment, 24 were ultimately excluded. The primary reasons for exclusion were: the lack of postnatal ASD outcome assessment (*n* = 9), imaging performed only in the neonatal or postnatal period (*n* = 1), insufficient methodological detail or case-report format (*n* = 5), purely animal-based studies (*n* = 2), overlapping cohorts (*n* = 2), and various other methodological inconsistencies (*n* = 5). After rigorous screening, eight studies met all eligibility requirements for inclusion in the qualitative synthesis. Due to considerable heterogeneity in MRI modalities, gestational age at scanning, outcome definitions, and statistical methods, a quantitative meta-analysis was not feasible. Thus, findings were synthesized narratively.

#### The Eligibility of Studies Was Guided by the Following Criteria

Original human studies; fetal magnetic resonance imaging (MRI) used to assess brain structure and/or function; postnatal ASD diagnosis or standardized measures of autistic traits; and either longitudinal or retrospective follow-up. Reviews, editorials, abstracts, animal studies, neonatal/postnatal-only MRI studies, and studies without ASD-related outcomes were excluded.

- Inclusion: original studies with human participants; use of fetal magnetic resonance imaging (MRI), both to assess brain structure and/or function; assessment of autism spectrum disorder (ASD) diagnosis after birth or use of standardized measures of autistic traits; and a study design that includes either long-term (longitudinal) or backwards-looking (retrospective) follow-up.
- Exclusion: Studies focusing only on neonatal or postnatal MRI, reviews, editorials, or abstracts, and studies lacking ASD-related outcomes.

For each selected article, we extracted key data points, including: primary author, publication year, and study design; sample size, gestational age at the time of MRI, and imaging modality (structural vs. rs-fMRI); targeted brain regions and neural networks; the clinical tools used for ASD assessment and primary statistical findings; adjustments made for potential confounding variables. In keeping with the PRISMA checklist accompanying this manuscript, a formal risk-of-bias tool was not applied because the review was narrative and the included evidence was highly heterogeneous.

Instead, methodological quality was appraised narratively by considering study design, sample size, ascertainment of outcomes, confounder adjustment, and possible sources of selection bias when interpreting the evidence. Given the wide variability in imaging parameters and clinical reporting, a narrative synthesis was chosen as the most robust way to present the integrated results. This approach was considered more appropriate than a formal systematic review with meta-analysis because the available evidence is sparse, methodologically diverse, and not amenable to quantitative pooling.

This PRISMA 2020 checklist is provided to document transparency in study identification, screening, selection, and reporting. The manuscript is framed as a PRISMA-informed narrative review rather than a formal systematic review or meta-analysis.

Accordingly, formal risk-of-bias assessment (Item 11) is not applicable, as the sparse and heterogeneous evidence base was synthesized qualitatively rather than quantitatively.

The electronic search strategy is reported in Appendix A of the manuscript.

## 3. Results

A total of eight studies met the eligibility criteria for this review (Table 1). The final selection encompasses a diverse range of methodologies, including structural fetal MRI cohorts, prospective resting-state fMRI (rs-fMRI) investigations, and studies focused on high-risk clinical populations, such as those with isolated ventriculomegaly or tuberous sclerosis complex (TSC). No formal meta-analysis was undertaken, and the resulting evidence base should be interpreted as exploratory and hypothesis-generating. To provide a broader neurodevelopmental context, the synthesis also incorporates translational animal research, imaging-genetics, and large-scale consortium data. Across these studies, gestational age at the time of MRI ranged from approximately 18 to 36 weeks, with postnatal follow-up periods extending from age two through to school age.

## 4. Structural Fetal MRI Findings

### 4.1. Prenatal Volumetric Alterations

In a retrospective clinical study, Ortug et al. [6] examined fetuses that later received an ASD diagnosis, identifying significant volumetric brain differences detectable between 18 and 36 weeks of gestation. The most reproducible finding was a marked increase in insular cortex volume in the ASD group. The insula plays a pivotal role in salience detection, emotional awareness, interception, and the integration of social information, domains that are frequently disrupted in individuals with ASD. Postnatal neuroimaging has long implicated altered insular function in the disorder, and the observation that volumetric enlargement may already be present in utero suggests that disruptions in cortical growth trajectories may begin earlier than previously thought. Beyond insular changes, the study also noted shifts in hemispheric asymmetry and regional volume distribution. These prenatal findings align with postnatal literature describing early brain overgrowth in infants later diagnosed with ASD, potentially extending the “overgrowth hypothesis” into the second and third trimesters of pregnancy. Further supporting these observations, Kim et al. [7] explored cortical morphology in preterm infants. Their work revealed that altered cerebral curvature, specifically within the orbitofrontal and cingulate regions, was associated with common genetic variants linked to ASD and lipid metabolism. While this study focused on the neonatal period, it provides compelling evidence that atypical cortical geometry may be an early marker of neurodevelopmental vulnerability tied to ASD related genetic pathways. The use of quantitative, MRI-based morphometric analysis in this context strengthens the idea that subtle structural deviations measured early in life are likely downstream manifestations of prenatal processes. At a systems level, the ENIGMA Working Group [3] found that cortical thickness alterations in ASD correlate with gene expression patterns involving pyramidal neurons, astrocytes, and microglia. These findings provide a vital biological framework for interpreting prenatal MRI anomalies, suggesting that structural differences detected in utero reflect underlying cellular and molecular shifts that continue to drive cortical maturation throughout development. Finally, the review by Amgalan et al. [1] highlights converging evidence for the prenatal origins of various neuropsychiatric conditions, including ASD. Their work underscores how mechanisms such as maternal immune activation, inflammation, hypoxia, and placental dysfunction can alter fetal brain development. Although their synthesis is narrative, it reinforces the biological plausibility that the structural and functional differences identified via fetal MRI represent the earliest observable manifestations of these complex prenatal influences.

### 4.2. High-Risk Structural Models: Ventriculomegaly and Tuberous Sclerosis Complex

Clinical conditions marked by identifiable prenatal structural anomalies offer a vital framework for understanding how fetal MRI can signal neurodevelopmental risks related to ASD. Specifically, the work of Hulshof et al. [8] and Kyriakopoulou et al. [9] centers on two distinct but conceptually aligned models: isolated fetal ventriculomegaly and tuberous sclerosis complex (TSC). In both cases, MRI-detectable abnormalities serve as early indicators of significantly increased ASD risk. Isolated fetal ventriculomegaly is among the most common central nervous system anomalies identified during routine prenatal screening. While ultrasound is typically the first to flag ventricular enlargement, fetal MRI is indispensable for refining the diagnosis and evaluating the health of the surrounding cortical mantle and white matter. Unlike simple measurements of ventricular size, MRI provides a nuanced assessment of cortical thickness, sulcation patterns, and white matter organization. Longitudinal studies in selected cohorts have reported that approximately 37.5% of children with isolated fetal ventriculomegaly meet ADOS-2 criteria for ASD by school age [9]. Beyond the higher prevalence of ASD, these children often exhibit challenges in sustained attention, working memory, and sensory processing. Notably, language delay in the preschool years has emerged as a strong predictor of a later ASD diagnosis, suggesting a clear developmental trajectory that links early structural variations to subsequent social communication difficulties. From a neurodevelopmental standpoint, ventricular enlargement may be a proxy for altered cortical growth dynamics, such as atypical white matter maturation or regional overgrowth. In this light, MRI is more than a confirmatory tool; it is a modality capable of placing ventricular changes within the broader context of brain maturation. Tuberous sclerosis complex offers a complementary high-risk model, one with a well-defined genetic etiology. In TSC, fetal MRI enables early detection and quantification of cortical tubers and subependymal lesions. Research has shown that the prenatal “lesion burden”, the extent of these structural abnormalities, correlates closely with cognitive delays and ASD diagnoses by age two. Interestingly, while epilepsy severity is a major factor in TSC, it was the prenatal cortical lesion load rather than seizure severity that more accurately predicted ASD outcomes. MRI is central to this clinical picture, as it enables precise anatomical characterization of cortical dysplasia and allows for objective lesion scoring while the fetus is still in the womb. Together, ventriculomegaly and TSC demonstrate that fetal MRI can identify structural deviations deeply linked to later neurodevelopmental outcomes. While these high-risk cohorts do not represent the general population, they provide convergent evidence that prenatal structural disruptions may signal the very beginning of an altered developmental path. These models strengthen the hypothesis that, for at least a subset of cases, ASD vulnerability originates from measurable disruptions in cortical development during fetal life.

## 5. Functional Fetal MRI Findings

The application of resting-state functional MRI (rs-fMRI) to the fetus is a relatively recent but transformative development in neuroimaging [10,11]. This technology allows us to observe the background communication between brain regions before they are even shaped by external postnatal experiences. In a prospective longitudinal study, Chen et al. [12] examined functional connectivity in utero and followed the cohort to assess autistic traits at age three. Their analysis revealed significant associations between prenatal connectivity patterns and later behavior. Specifically, higher scores on autistic traits were associated with atypical connectivity across several key systems: the cingulate–temporal and prefrontal–opercular networks, as well as the visual–prefrontal and cerebellar–opercular circuits.

A notable pattern emerged in which higher autistic traits correlated with unusually strong connectivity within certain socio-emotional networks, whereas reduced connectivity was observed involving the cerebellum. These findings are noteworthy given the roles of the regions involved. The cingulate cortex is a hub for social cognition and emotional regulation, while prefrontal regions govern executive control and social reasoning. Furthermore, the cerebellum, once thought to be primarily involved in motor coordination, is now increasingly recognized for its vital role in social and cognitive processing, with both structural and functional abnormalities frequently documented in ASD [13]. The fact that these connectivity differences are detectable during fetal life may indicate that the architecture of large-scale brain networks may begin to diverge from typical trajectories well before birth. These results are consistent with the idea that the functional wiring of the brain in ASD is altered at a very fundamental level, preceding the emergence of overt behavioral symptoms.

## 6. Translational Evidence: Maternal Autoantibody Model

To better understand the biological mechanisms behind these observations, translational research has utilized experimental models to bridge the gap between prenatal exposure and brain morphology. Bruce et al. [14] developed a mouse model focused on maternal autoantibody-related ASD, revealing striking neuroanatomical changes in offspring exposed to these antibodies in utero. High-resolution MRI analysis of these models demonstrated not only an increase in total brain volume but also the specific enlargement of various cortical and subcortical structures. Furthermore, structural covariance analysis pointed toward a desynchronization at the network level, with female offspring showing particularly significant volumetric shifts. While we must be cautious when extrapolating findings from rodents to humans, this model offers critical mechanistic support for how immune-mediated prenatal influences can reshape brain growth and network architecture. These results are highly consistent with human epidemiological data that link maternal immune activation (MIA) to an increased risk of ASD, fitting neatly into broader theoretical frameworks that emphasize the biological vulnerability of the developing fetus [1].

## 7. Discussion

When viewed as a whole, the structural and functional evidence gathered in this review points toward a relatively consistent emerging narrative: the neurodevelopmental divergence associated with ASD may begin during fetal life [4,5]. A recurring theme in the structural literature is prenatal volumetric enlargement, particularly in the insular cortex. This finding provides support for the “early brain overgrowth hypothesis,” which posits that atypical increases in regional brain volume result from fundamental disruptions in neurodevelopment, such as dysregulated neuronal proliferation, excessive synaptic density, or a failure of pruning mechanisms that normally refine neural connections.

The identification of these volumetric shifts in utero should be interpreted as associative rather than causal evidence, suggesting that deviations in cortical growth trajectories may be early imaging correlates of later neurodevelopmental outcomes rather than definitive proof of causation. Functional MRI findings complement this structural data, revealing that atypical organization within large-scale brain networks is already evident before birth. The altered connectivity identified within the cingulate, prefrontal, temporal, and cerebellar circuits suggests that ASD may be conceptualized as a disorder of early network integration rather than as an isolated regional defect. Because these specific networks are the engines of social cognition, executive function, salience processing, and sensorimotor integration, their prenatal dysregulation may provide a plausible neurobiological foundation for the social communication challenges and restricted behaviors seen later in life. The case for a prenatal origin of ASD vulnerability is further strengthened by the study of high-risk structural conditions, such as isolated ventriculomegaly and tuberous sclerosis complex [8,9]. In these specific clinical contexts, structural abnormalities detectable via fetal MRI are tied to significantly higher rates of ASD or broader neurodevelopmental impairment. These models reinforce the concept of a developmental continuum, in which measurable structural deviations in the womb are linked to postnatal functional and behavioral outcomes. In this emerging framework, MRI plays a pivotal role. It enables the identification and characterization of subtle cortical and network-level anomalies during a phase of life previously considered a black box. While the current body of evidence is not yet robust enough to support general population screening, these findings suggest that fetal MRI is becoming a valuable tool for assessing neurodevelopmental vulnerability in high-risk contexts and for deepening understanding of the early pathogenesis of ASD.

## 8. Strengths and Limitations of Included Studies

### 8.1. Strengths

Several core methodological strengths emerge from the literature analyzed in this review. Chief among these is the longitudinal design adopted by most studies, utilizing either prospective or retrospective follow-up. This temporal depth is crucial because it allows for a more meaningful association between prenatal MRI findings and later clinical outcomes, providing a level of causal plausibility that cross-sectional designs cannot. The reliability of these findings is further bolstered by the use of standardized and validated diagnostic tools. By relying on established instruments such as the Autism Diagnostic Observation Schedule (ADOS-2) or rigorous quantitative trait scales, these studies minimize the risk of diagnostic misclassification and enhance the reproducibility of their results. From a technical perspective, the research reviewed here sits at the cutting edge of prenatal neuroscience. The shift from basic structural assessments to high-resolution volumetric segmentation and resting-state functional MRI (rs-fMRI) represents a significant leap forward. These techniques have allowed researchers to map regional brain growth and emerging large-scale connectivity networks, providing a previously unavailable granular view of development. Importantly, the findings show a remarkable biological coherence with known ASD neurobiology. The observed alterations are not random; they consistently converge on regions and networks, such as the insular cortex, cingulate areas, prefrontal networks, and the cerebellum, that are hallmarks of the ASD brain in postnatal life. This alignment enhances the construct validity of fetal MRI, suggesting that these early deviations are indeed the first markers of an atypical developmental trajectory. Finally, the focus on high-risk clinical populations, such as those with isolated ventriculomegaly or tuberous sclerosis complex (TSC), provides a natural context to observe how identifiable structural disruptions can lead to elevated ASD risk, reinforcing the concept of a neurodevelopmental continuum that begins long before birth.

### 8.2. Limitations

Despite these promising insights, several significant limitations must be considered when interpreting the data. The most pressing issue is the small sample size across nearly all included studies. Many cohorts consisted of fewer than 40 participants, with the number of confirmed ASD cases often falling below 15. These limited numbers inherently reduce statistical power, increase the risk of Type I and Type II errors, and make it difficult to establish stable effect size estimates. Accordingly, any proposed prenatal biomarker should currently be regarded as preliminary, non-validated, and insufficiently generalizable for clinical prediction outside selected research settings. Selection bias also remains a concern. Because many participants were recruited from tertiary referral centers or specialized fetal medicine units, the samples are likely enriched for more complex or high-risk cases. While this provides valuable data on those specific conditions, it limits the extent to which findings can be generalized to the broader obstetric population and may inadvertently overestimate the strength of certain associations. The field also struggles with substantial heterogeneity in MRI protocols. Differences in scanner field strength, pulse sequences, motion-correction techniques, and segmentation pipelines, coupled with a wide range of gestational ages at the time of scanning (18–36 weeks), create significant methodological variability. Without standardized frameworks for acquisition and processing, comparing results across different centers remains a challenge. Furthermore, variability in postnatal assessment adds another layer of complexity. Follow-up ages ranged from early toddlerhood to school age, and diagnostic procedures were not uniform. Since ASD symptoms can evolve significantly in early childhood, these differences in timing and criteria may impact the reported prevalence and classification accuracy. Finally, the control of confounding variables was inconsistent. Many studies did not fully account for critical factors such as maternal psychiatric history, genetic predisposition, or socioeconomic status, all of which can independently influence neurodevelopment. Moreover, the heavy reliance on high-risk populations like those with TSC or ventriculomegaly means that these findings may not fully capture the diverse spectrum of ASD risk as it appears in unselected, general pregnancies. At the review-process level, additional limitations include the narrative design itself, the absence of a formal quantitative risk-of-bias tool, and the possibility that relevant unpublished or non-indexed evidence was not captured despite the structured search strategy.

## 9. Clinical and Research Implications

At this stage, fetal MRI should not be viewed as a predictive tool for the general population. However, its value within high-risk cohorts is becoming increasingly clear. In these specific cases, prenatal imaging can play a vital role in informing neurodevelopmental counseling, enhancing postnatal surveillance, and building more nuanced risk-stratification frameworks. Looking ahead, the research community must prioritize large-scale, multicenter prospective cohorts and the standardization of both imaging protocols and ASD diagnostic assessments. The future of the field lies in integrating genetic, epigenetic, and environmental risk markers, as well as in developing sophisticated machine learning models to synthesize this complex data.

### 9.1. Future Directions

The evolution of fetal MRI from a descriptive clinical observation to a predictive biomarker platform rests on two emerging pillars: the integration of Artificial Intelligence (AI) and the advancing field of Imaging-Genetics.

### 9.2. Artificial Intelligence and Radiomics

Traditional clinical evaluations of fetal MRI are often hindered by the limits of human visual perception and the persistent challenge of fetal motion artifacts. Today, the implementation of Deep Learning (DL) algorithms, specifically convolutional neural networks (CNNs), is transforming the field by enabling automated, high-precision segmentation of fetal brain structures. Beyond basic volumetry, the rise of Radiomics enables researchers to extract high-dimensional quantitative data, including voxel-level texture, signal-intensity gradients, and subtle morphological features imperceptible to the naked eye. When integrated into machine learning models, these hidden prenatal markers can help generate predictive risk scores, potentially identifying atypical developmental paths long before behavioral symptoms appear. At the same time, evidence remains early: most available fetal MRI AI studies focus on automated brain extraction, motion correction, reconstruction, tissue segmentation, or anomaly classification rather than on validated prediction of later ASD. For this reason, AI and radiomics should presently be viewed as enabling technologies that may improve measurement precision and future risk stratification, rather than as established predictive tools for autism in clinical practice.

### 9.3. Neuro-Transcriptomics and Imaging-Genetics

The biological validity of fetal MRI findings is increasingly bolstered by imaging-genetics, a field that bridges the gap between macroscopic brain changes and microscopic gene expression. By referencing MRI data with spatiotemporal gene expression atlases, researchers have discovered that regions showing atypical growth in ASD fetuses, such as the insular cortex, overlap significantly with areas where ASD-linked risk genes are most active during the second and third trimesters (Table 2). This neuro-transcriptomic lens suggests that prenatal volumetric enlargement is the macroscopic sign of dysregulated molecular events, such as excessive neuronal proliferation or a failure in synaptic pruning. This bridge between imaging and genetics provides the necessary biological context to turn an interesting observation into a reliable clinical marker (Table 3).

### 9.4. Peripheral Biomarkers of Neurodevelopmental Trajectories: The Case of Neurofilament Light Chain

Emerging translational evidence suggests that circulating neurofilament light chain (NfL), a sensitive blood biomarker of neuroaxonal integrity measurable through ultrasensitive platforms [15], may capture aspects of altered axonal dynamics in autism spectrum disorder (ASD). Several cross-sectional pediatric studies have reported higher serum NfL concentrations in children with ASD compared with typically developing controls, with some investigations describing associations between elevated NfL levels and greater symptom severity [16,17]. However, findings remain inconsistent. Other cohorts have observed no significant group differences or even inverse correlations between NfL levels and specific behavioral domains [18,19]. Such discrepancies likely reflect heterogeneity in assay platforms, cohort size, age distribution, and clinical phenotyping. Taken together, current evidence does not support a neurodegenerative interpretation of NfL alterations in ASD. Rather, NfL may reflect dynamic processes of axonal remodeling and network maturation within atypical neurodevelopmental trajectories. Robust longitudinal studies are now required to determine whether peripheral NfL tracks developmental change, symptom progression, or treatment response across childhood. In a future multimodal framework, fetal MRI could provide early structural and functional signatures of altered brain development, whereas peripheral biomarkers such as NfL might offer a longitudinal postnatal readout of ongoing neuroaxonal remodeling.

Their convergence would therefore not be simultaneous at the same developmental window, but complementary across time, potentially linking prenatal imaging phenotypes with postnatal biological trajectories.

### 9.5. Multimodal Predictive Integration: Imaging and Peripheral Biomarkers

If fetal MRI reveals early structural and functional divergence, peripheral biomarkers may provide a complementary postnatal biological readout of how these trajectories unfold over time. The future of prenatal ASD research therefore lies not in single-modality discovery, but in the systematic integration of advanced neuroimaging analytics with molecular markers across developmental stages [2,10,12]. AI-driven radiomics and machine learning approaches already enable the extraction of high-dimensional fetal MRI features beyond conventional volumetric metrics [10,11]. When coupled with imaging-genetics frameworks linking cortical morphology to spatiotemporal gene expression patterns [3], and with longitudinal peripheral markers such as NfL, these data streams could converge into multimodal risk models. By identifying latent patterns across imaging, genetic architecture, and circulating biomarkers, machine learning frameworks could move the field beyond descriptive association toward individualized, developmentally informed risk stratification. In this integrated perspective, fetal MRI and peripheral biomarker dynamics are not competing signals, but complementary layers within a unified systems-level model of ASD pathogenesis [1,3,4].

## 10. Conclusions and Future Perspectives

The evidence gathered in this review underscores a fundamental shift in our understanding of autism spectrum disorder: the structural and functional brain divergence associated with ASD may not be solely a postnatal phenomenon. Key indicators, such as insular volumetric expansion, atypical cortical folding, and early network dysregulation, collectively point toward the prenatal period as a potentially critical window in the pathogenesis of the disorder. In this context, we strongly support future studies aiming at further investigating fetal neurodevelopmental trajectories and their deviations. Fetal MRI provides an unprecedented in vivo lens, allowing us to witness the very earliest chapters of neurodevelopmental trajectories. Moving from research observations to clinical implementation requires us to evolve beyond qualitative assessment. The ultimate objective of prenatal research now lies in the synergy of a multimodal framework. This approach seeks to match AI-driven Radiomics, which can identify subtle imaging signatures that escape the human eye, with neuro-transcriptomics, which bridges the gap between these macroscopic changes and their genetic risk profiles. By integrating this high-dimensional data with environmental factors, such as maternal immune activation, fetal MRI may eventually contribute to early risk-stratification frameworks. Ultimately, while large-scale longitudinal validation is essential to confirm these preliminary biomarkers, this integrated approach holds the power to transform our understanding of ASD. It paves the way for a future in which we can identify neurodevelopmental vulnerability at its source, opening the door to early prenatal or postnatal neuroprotective interventions once thought impossible.

## Figures and Tables

**Table 1:** Prisma checklist.

Section	Item	PRISMA Item Description	Location in Manuscript
Title	1	Identify the report as a systematic review or review.	Title
Abstract	2	Provide a structured summary including background, objectives, methods, results, and conclusions.	Abstract
Introduction	3	Describe the rationale for the review in the context of existing knowledge.	Introduction
	4	Provide an explicit statement of the objectives or questions addressed by the review.	Introduction
Methods	5	Specify inclusion and exclusion criteria for the review.	Methods—Study Selection
	6	Specify all databases, registers, and other sources searched.	Methods—Search Strategy
	7	Present the full search strategy, including keywords and Boolean operators.	Methods—Search Strategy
	8	Describe the process used to determine study eligibility.	Methods—Study Selection
	9	Describe the data extraction process.	Methods
	10	List and define all outcomes or variables extracted from included studies.	Methods
	11	Describe the methods used for risk of bias assessment (if applicable).	Not applicable (narrative review)
	12	Describe methods used for data synthesis.	Methods
Results	13	Describe the study selection process, preferably with a flow diagram.	Results/Figure 1
	14	Present characteristics of included studies.	Results/Table 1
	15	Present results of individual studies.	Results
	16	Present results of the synthesis of included studies.	Results
Discussion	17	Provide a general interpretation of results in context of other evidence.	Discussion
	18	Discuss limitations of the evidence included in the review.	Strengths and Limitations
	19	Discuss limitations of the review process.	Strengths and Limitations
Other Information	20	Describe sources of funding for the review.	Funding Statement
	21	Declare competing interests.	Conflicts of Interest

**Table 2:** Summary of studies on MRI findings and ASD.

Title	First Author	Journal	Sample Size	Study Design	Setting	Main Findings
Prenatal origins of neuropsychiatric diseases	Amgalan (2021) [1]	Acta Paediatrica	Narrative review (no direct sample)	Literature review	George Washington University/Children’s National, USA	Evidence for prenatal origins of ASD and other psychiatric disorders. Involved mechanisms include infection, inflammation, hypoxia, maternal stress and placental dysfunction. Growing role of advanced fetal MRI.
Autism-associated brain differences can be observed in utero using MRI	Ortug (2024) [6]	Cerebral Cortex	39 fetuses (9 pASD; 30 controls) between 18–36 weeks	Retrospective study with fetal MRI volumetric segmentation	Boston Children’s Hospital (Harvard), USA	Identification of fetal volumetric biomarkers associated with ASD. Increased insular volume proposed as an early prenatal biomarker.
Altered Cerebral Curvature in Preterm Infants Is Associated with the Common Genetic Variation Related to Autism Spectrum Disorder and Lipid Metabolism	Kim (2022) [7]	Journal of Clinical Medicine	Preterm infants (imaging-genetics study; N reported in original article)	Imaging-genetics study with neonatal MRI at term-equivalent age	Hanyang University/Seoul National University, Korea	Altered cortical curvature (orbitofrontal and cingulate regions) associated with genetic variants (OXTR, FADS2, COMT) related to ASD and lipid metabolism. Highlights genetic vulnerability in early cortical maturation.
Fetal functional connectivity prospectively associates with autistic traits in toddlerhood	Chen (2026) [12]	NeuroImage: Clinical	62 fetuses with 3-year follow-up	Prospective longitudinal study with fetal rs-fMRI	New York University Grossman School of Medicine, USA	Fetal functional connectivity (cingulate-temporal, prefrontal–operculum networks) associated with autistic traits at age 3. First prospective evidence of behavioral prediction using fetal rs-fMRI.
Fetal Brain MRI Findings Predict Neurodevelopment in Children with Tuberous Sclerosis Complex	Hulshof (2021) [8]	Journal of Pediatrics	41 children with TSC	Retrospective multicenter study (EPISTOP consortium)	European EPISTOP centers	Fetal MRI lesion score correlated with cognitive, motor development and ASD diagnosis at 2 years. Prenatal cortical lesions predicted neurodevelopmental outcome but not epilepsy severity.
Virtual Histology of Cortical Thickness and Shared Neurobiology in 6 Psychiatric Disorders	Writing Committee ENIGMA Working Groups (2020) [3]	JAMA Psychiatry	12,721 cases; 15,600 controls (ASD + 5 disorders)	Multicenter ENIGMA mega-analysis with T1 MRI and gene expression analysis	International ENIGMA Consortium	Cortical thickness differences in ASD associated with gene expression of pyramidal cells, astrocytes and microglia. Shared prenatal (neurodevelopmental) and postnatal (synaptic plasticity) mechanisms identified.
Characterisation of ASD traits among a cohort of children with isolated fetal ventriculomegaly	Kyriakopoulou (2023) [9]	Nature Communications	24 children with isolated ventriculomegaly; 10 controls	Longitudinal observational study with school-age follow-up	King’s College London, UK	37.5% of children with ventriculomegaly exceeded ADOS-2 threshold for ASD. Preschool language delay predicted ASD symptoms. Association between fetal structural alterations and autistic traits.
Sexually dimorphic neuroanatomical differences relate to ASD-relevant behavioral outcomes in a maternal autoantibody mouse model	Bruce (2021) [14]	Molecular Psychiatry	22 MAR-ASD mice (11 M, 11 F) and 23 controls (12 M, 11 F)	Experimental animal study with high-resolution ex vivo MRI (7T)	University of California Davis, USA	Increased total brain volume and enlargement of 31 regions in MAR-ASD mice, especially in females. Network alterations and correlation between regional overgrowth, social deficits and repetitive behaviors.

**Table 3:** Integration of Fetal MRI Biomarkers and Genetic-Biological Correlates.

Fetal MRI Biomarker	Associated Brain Region	Potential Genetic/Cellular Mechanism	Key ASD Related Processes
Increased Volumetric Growth	Insular Cortex, Frontal Lobe	Overexpression of Proliferation Genes (e.g., *PTEN* pathway)	Accelerated neuronal production; failure of early apoptosis.
Atypical Functional Connectivity	Cingulate & Prefrontal Networks	Dysregulation of Synaptogenesis Genes (e.g., *SHANK3*, *NLGN3*)	Altered formation of long-range excitatory/inhibitory balance.
Altered Cortical Folding/Curvature	Orbitofrontal & Temporal Regions	Disruptions in Neuronal Migration Genes (e.g., *RELN*, *CNTNAP2*)	Abnormal laminar organization and cortical patterning.
Subependymal Nodules/Tubers	Periventricular Zones	*TSC1/TSC2* Gene Mutations	Dysregulated mTOR signaling leading to focal cortical dysplasia.

## Data Availability

No new data were created or analyzed in this study.

## Appendix A. Electronic Search Strategy

The following keyword strategy was used across MEDLINE, Embase, Scopus, and Web of Science, with syntax adapted to the requirements of each database:

(“fetal” OR “foetal” OR “prenatal” OR “antenatal” OR “in utero”) AND (“magnetic resonance imaging” OR MRI OR “fetal MRI” OR “functional MRI” OR fMRI OR rs-fMRI) AND (autism OR “autism spectrum disorder” OR ASD OR “autistic traits” OR neurodevelopment*). Supplementary backward citation tracking was performed by screening the reference lists of eligible studies and relevant review articles. The search covered records from database inception through January 2026, with no language restrictions.
